# The *Hexokinase 1* 5′-UTR Mutation in Charcot–Marie–Tooth 4G Disease Alters Hexokinase 1 Binding to Voltage-Dependent Anion Channel-1 and Leads to Dysfunctional Mitochondrial Calcium Buffering

**DOI:** 10.3390/ijms25084364

**Published:** 2024-04-15

**Authors:** Maria Ceprian, Raul Juntas-Morales, Graham Campbell, Ulrike Walther-Louvier, François Rivier, William Camu, Florence Esselin, Andoni Echaniz-Laguna, Tanya Stojkovic, Françoise Bouhour, Philippe Latour, Nicolas Tricaud

**Affiliations:** 1 Institute for Neuroscience of Montpellier (INM), University Montpellier, INSERM, 34091 Montpellier, France; maria.ceprian@achucarro.org (M.C.);; 2 Clinique du Motoneurone, Explorations Fonctionnelles Neurologiques, Service de Neurologie, Hôpital Universitaire Gui de Chauliac, 34295 Montpellier, France; raul.juntas@vallhebron.cat (R.J.-M.); w-camu@chu-montpellier.fr (W.C.); f-esselin@chu-montpellier.fr (F.E.); 3 Unidad Neuromuscular, Servicio de Neurologia, Hospital Universitario Vall d’Hebron, 08035 Barcelona, Spain; 4 Service de Neuropediatrie, Hôpital Universitaire Gui de Chauliac, 34295 Montpellier, France; u-louvier@chu-montpellier.fr (U.W.-L.); f-rivier@chu-montpellier.fr (F.R.); 5 AEL, Department of Neurology, Bicetre University Hospital, Paris Saclay University, 94270 Paris, France; andoni.echaniz-laguna@aphp.fr; 6 Service de Neurologie, Hôpital Universitaire Pitié-Salpêtrière, 75013 Paris, France; tanya.stojkovic@aphp.fr; 7 Service de Neurologie, Hôpital Universitaire Lyon, 69500 Lyon, France; francoise.bouhour@chu-lyon.fr; 8 Centre de Biologie Est Biochimie et Biologie Moléculaire, Hospices Civils de Lyon, 69677 Bron, France; philippe.latour@chu-lyon.fr; 9 I-Stem, UEVE/UPS U861, INSERM, AFM, 91100 Corbeil-Essonnes, France

**Keywords:** CMT4G, mitochondria, Hexokinase I, VDAC

## Abstract

Demyelinating Charcot–Marie–Tooth 4G (CMT4G) results from a recessive mutation in the 5′UTR region of the Hexokinase 1 (HK1) gene. HK participates in mitochondrial calcium homeostasis by binding to the Voltage-Dependent Anion Channel (VDAC), through its N-terminal porin-binding domain. Our hypothesis is that CMT4G mutation results in a broken interaction between mutant HK1 and VDAC, disturbing mitochondrial calcium homeostasis. We studied a cohort of 25 CMT4G patients recruited in the French gypsy population. The disease was characterized by a childhood onset, an intermediate demyelinating pattern, and a significant phenotype leading to becoming wheelchair-bound by the fifth decade of life. Co-IP and PLA studies indicated a strong decreased interaction between VDAC and HK1 in the patients' PBMCs and sural nerve. We observed that either wild-type HK1 expression or a peptide comprising the 15 aa of the N-terminal wild-type HK1 administration decreased mitochondrial calcium release in HEK293 cells. However, mutated CMT4G HK1 or the 15 aa of the mutated HK1 was unable to block mitochondrial calcium release. Taken together, these data show that the CMT4G-induced modification of the HK1 N-terminus disrupts HK1-VDAC interaction. This alters mitochondrial calcium buffering that has been shown to be critical for myelin sheath maintenance.

## 1. Introduction

Together with CMT4D, CMT4G disease is the most common CMT subtype affecting the gypsy population from Bulgaria, the Czech Republic, Slovakia, and Spain [1,2,3,4,5]. This disease is more severe than CMT1A, the most frequent CMT disease, with an onset before the age of 15 years old for all patients. Its clinical phenotype is a severe motor and sensory neuropathy with prominent distal muscle atrophy, that spreads to the proximal lower limbs in early adulthood. Nerve conduction studies show demyelinating features and a diffuse absence of sensory potentials even in patients tested in the first years of life. Genetically, CMT4G results from a recessive mutation in the 5′ non-coding sequence of the Hexokinase 1 (HK1) gene. This gene encodes the first enzyme of glycolysis that phosphorylates glucose into glucose-6 phosphate [6]. This protein is critical as HK1 mutations in humans and in mice result in a severe non-spherocytic hemolytic anemia [7,8] due to the loss of the metabolic activity of the enzyme. However, the CMT4G mutation does not affect the enzymatic activity of HK1 or its level of expression [5]. Thus, the pathomechanism of CMT4G remains unclear.

In cells, a large amount of HK is associated with mitochondria [9]. Indeed, HK interacts through its N-terminal porin binding domain (PBD) with the Voltage-Dependent Anion Channel (VDAC), a porin of an outer mitochondrial membrane [10]. This interaction controls the exit of calcium and ATP from the mitochondria through VDAC [11].

The control of VDAC permeability to calcium is critical for cells. At the plasma membrane, calcium ion flux depolarizes the cell, inducing depolarization signaling along the membrane or an action potential in excitable cells. In the cytoplasm, several proteins use calcium ions as a cofactor such as calmodulin or troponin. To control the calcium amount in the cytoplasm, cells store a high amount of this ion in several organelles such as the endoplasmic reticulum (ER) or mitochondria. Therefore, the release of calcium from these organelles is a key event that is strictly controlled by the cell. As an example, the massive release of mitochondrial calcium is the triggering event of programmed cell death [12], while a moderated release activates the cell differentiation mechanism [12,13]. VDAC permeability to calcium is therefore finely tuned and HK is its main natural inhibitor [6]. The N-terminus of HK is responsible for its interaction with VDAC [10], and peptides that mimic this part of the molecule are able to block mitochondrial calcium release through VDAC [14,15,16]. In addition, methyl jasmonate (MJ), a plant stress hormone that specifically binds to HK, changes its conformation and breaks its interaction with VDAC [17,18]. In some cancer cells, this induces a massive release of mitochondrial calcium and cell death [19].

We have recently shown in myelinating Schwann cells (mSCs) of mouse peripheral nerve that a moderate mitochondrial calcium release through VDAC is one of the first steps that leads to demyelination [11]. This mechanism is involved in traumatic demyelination but also in diabetic peripheral neuropathy in mice [11], suggesting that alteration of the VDAC permeability to calcium is involved in different forms demyelinating peripheral neuropathies.

We therefore hypothesized that CMT4G mutation induced a molecular change in HK that leads to an alteration of its binding to VDAC and a change in mitochondrial calcium homeostasis in mSCs. According to the literature, this would promote the demyelination of mSCs.

## 2. Results

### 2.1. Mimicking CMT4G in HEK293T Cells Reveals a Defect in HK1/VDAC Interaction

**Figure 1:**
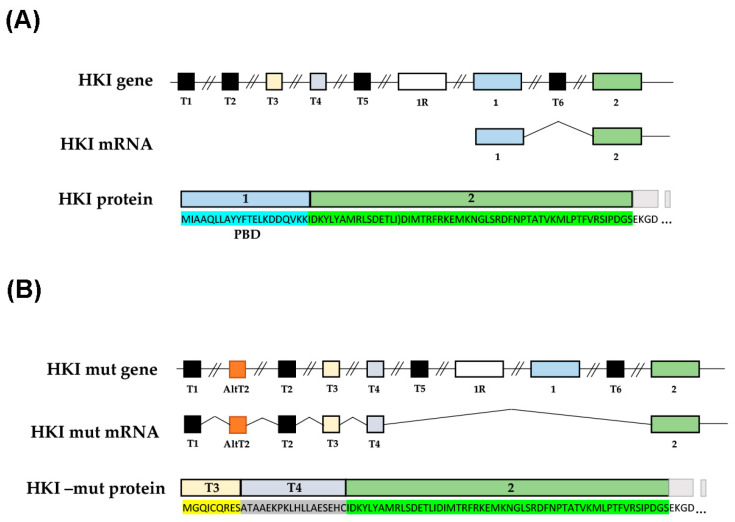
**Molecular basis of the alternative splicing of HK1 mRNA proposed in CMT4G.** Schematic structure of the N-terminal part of the HK1 gene, mRNA, and protein in wild-type condition (**A**) and in CMT4G mutant condition (**B**) according to Hantke et al., 2009 [5]. Boxes show exons in the gene, alternatively spliced exons in the mRNA, and translated domains in the protein. The amino acid (aa) composition of each translated domain is highlighted in color. Exon 1 is translated in 21 aa. Exons T3 and T4 are translated in 9 and 18 aa, respectively.

Previous analysis of the genetic defect in CMT4G patients [5] suggested that cells produce a mutant mRNA with an alternative splicing AltT2 (Figure 1). This novel splicing modifies the first translated exon of HK1 protein into the T3 and T4 alternative exon (Figure 1B). This modifies the amino acid (aa) composition of the protein up to aa 21 and adds 6 aa to the overall number of the HK1 sequence (Figure 1B). As HK binds to VDAC through its N-terminus, we hypothesized that this alteration of the HK1 N-terminus has a significant effect on this interaction.

**Figure 2:**
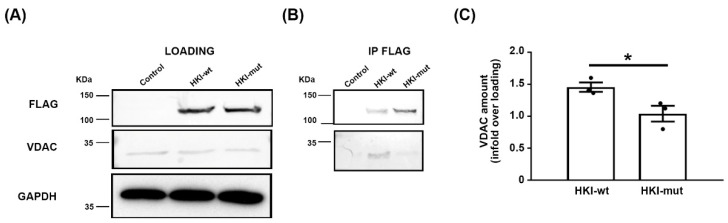
**Mutant HK1 does not coimmunoprecipitate with VDAC in transfected cells.** (**A**) Representative WB of protein extracts (10 mg/mL) before immunoprecipitation. HKI-wt and HKI-mut were detected using antibody against the Flag tag (upper panel); WB for GAPDH is used as a loading control (lower panel). (**B**) Representative WB of immunoprecipitated proteins with Flag antibody (IP FLAG) hybridized with Flag (upper panel) or VDAC (lower panel) antibodies. The same amount of antibody was used for each immunoprecipitation and the stronger band for HKI-mut is not representative. (**C**) VDAC amount was quantified in immunoprecipitates and normalized on VDAC amount in protein extracts before immunoprecipitation. n = 3 independent experiments. Each dot the value of an independent experiment. Error bars show SEM. Statistical test shows two-tailed Student *t*-test. * *p* < 0.05; *p*-value = 0.0447.

In order to analyze how the alternative splicing promoted by the mutation affects the HK-VDAC interaction, we first mimicked this mechanism in vitro. We expressed in HEK293T cells wild-type (wt) HK1 (HKI-wt) or a mutation-induced alternatively spliced HK1 isoform (HKI-mut), both tagged in the C-terminal with a Flag tag. For an identical amount of protein extract, HKI-wt and HKI-mut were expressed at a similar level in transfected cells and no difference in molecular weight was detected (Figure 2A upper panel). VDAC was also expressed at similar levels in cells expressing either HK1 isoforms and in control cells (Figure 2A lower panel). Next, we immunoprecipitated each HK1 isoform using an anti-Flag antibody and we analyzed the amount of VDAC in the precipitate using Western blotting. While both HK1 isoforms were immunoprecipitated with Flag antibody (Figure 2B upper panel), significantly less VDAC co-precipitated with HKI-mut than with HKI-wt (Figure 2B lower panel, quantification in Figure 2C). This indicated that the alteration of the 21 aa of the HK1 N-terminus was sufficient to jeopardize its interaction with VDAC.

### 2.2. Defect in HK1/VDAC Interaction Results in the Alteration of Mitochondrial Calcium Homeostasis

**Figure 3:**
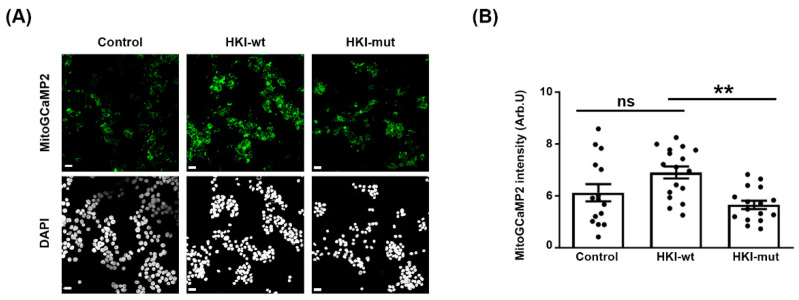
**HK1 and mutant HK1 have opposite effects on calcium homeostasis in mitochondria.** HEK293 cells were transfected with a plasmid expressing mitoGCaMP2 alone (control) or together with plasmids expressing HKI-wt or HKI-mut. A period of 48 h after transfection, cells were fixed and imaged for mitoGCaMP2. (**A**) Representative images of cells expressing only the probe or the probe plus HKI-wt or HKI-mut. Scale bars show 50 µm. (**B**) The mean intensity value per area was measured in 20 ROIs containing several cells. Then, the background intensity measured in empty areas of each ROI was subtracted to the value of each ROI. Error bars show SEM. Statistical test shows one-way ANOVA by Bonferroni’s multiple comparison post hoc test. ** *p* < 0.01 *p*-value = 0.0029. A total of 3 independent experiments were performed.

As HK controls mitochondrial calcium homeostasis through its interaction with VDAC, we therefore investigated whether mitochondrial calcium homeostasis was altered in cells over-expressing HKI-wt or HKI-mut. We expressed either isoform together with a fluorescent calcium sensor probe targeted to mitochondrial matrix, mito-GCaMP2, in HEK293 cells [16]. This probe detects mitochondrial calcium levels in living cells as previously shown in vitro and in vivo [11,20,21]. Over-expressing HKI-wt resulted in slightly more fluorescence (Figure 3A), suggesting an increased amount of calcium in mitochondria. However, expressing HKI-mut led to a decrease, resulting in a significant opposite effect of the two isoforms on mitochondrial calcium (Figure 3A, quantified in Figure 3B). These data suggested that an excess of wt HK1 blocked calcium flux out of the mitochondria through VDAC, while mutant HK1, which does not bind to VDAC, allowed calcium to flow.

### 2.3. N-Terminus Peptide of Mutant HK1 Lacks the Ability to Block Mitochondrial Calcium Release through VDAC

**Figure 4:**
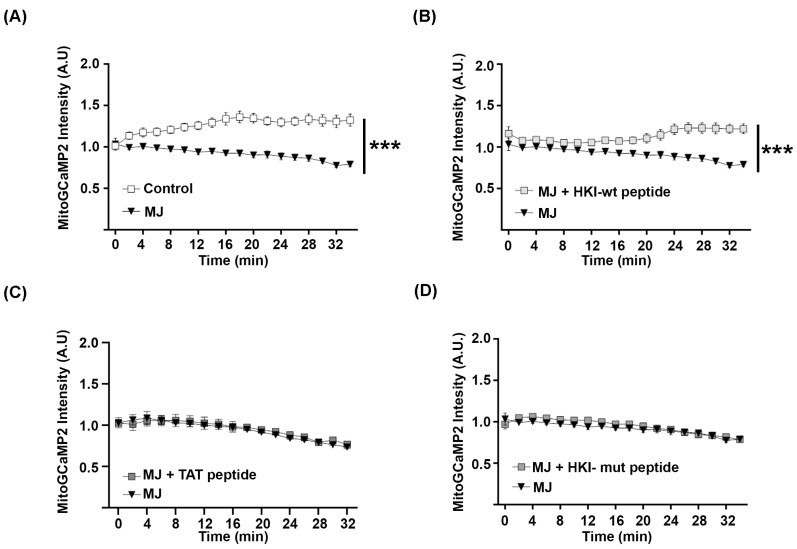
**HK1-wt but not mutated N-terminal peptide blocks release of calcium from mitochondria.** HEK293 cells expressing mitoGCaMP2 were not treated (control) (**A**) or treated with 6 mM methyl jasmonate (MJ) and 5 µM peptides at t = 0 (**B**–**D**). Cells were imaged and fluorescence was measured at = 0 min, 2 min, and then every 4 min until 34 min. The mean intensity value per area was measured in 20 ROIs containing several cells. Then, the background intensity measured in empty areas of each ROI was subtracted by the value of each ROI. Error bars show SEM. Statistical test shows two-way ANOVA. *** *p* < 0.001; *p*-value < 0.001. n = 3 independent experiments, 2 replicas per experiment.

In order to confirm that mutant HK1 is unable to control mitochondrial calcium release, we next investigated the function of the HK1 N-terminal peptide that mediates its interaction with VDAC. MJ was used to displace HK1 from VDAC in HEK293 cells expressing mitoGCaMP2. This induced a steady decrease in mitochondrial calcium over time (Figure 4A). When these cells were treated both with MJ and a peptide bearing the 15 N-terminal aa of HKI-wt (MAAQLLAYYFTELK) fused to a TAT cell-penetrating peptide (CPP; GRKKRRQRRRPPQ), the mitochondrial calcium level did not decrease (Figure 4B). The TAT peptide alone did not prevent the decrease in mitochondrial calcium induced by MJ (Figure 4C), confirming that the HKI-wt peptide blocked calcium release out of mitochondria.

We next tested the impact of a peptide bearing the 15 N-terminal aa of HK-mut (MGQICQRESATAAEK) fused with TAT. We found that this peptide had no effect on the mitochondrial calcium release induced by MJ (Figure 4D). So, we conclude that the alteration of the N-terminus of mutant HK1 prevents its interaction with VDAC and impairs its function as a VDAC inhibitor.

### 2.4. Characterization of a Novel CMT4G Patient Series in the French Gispy Population

We collected clinical and electrophysiological features of 25 patients belonging to nine unrelated families of the French gypsy community diagnosed with CMT4G (Table 1). All probands were homozygous for substitution g.9712G > C in the HK1 (NM_033498) gene according to the previously described genotype for CMT4G [4].

The male-to-female ratio was 3/1 and the age ranged from 3 to 70 years. The median age of onset of lower limb weakness was 7.4 years, while the hand phenotype onset was between the second and fourth decades. Muscle weakness worsened with age and all patients older than 50 years were wheelchair-bound. Most of the patients had foot deformities, usually pes cavus but also clawing of the toes. Nine of them required ankle arthrodesis. Spinal deformity and deafness were not observed. All patients showed distal sensory loss and areflexia. Fourteen patients determined their CMTNS, with a median score of 15/36. Electrophysiological recordings were available for 15 patients only in the upper limbs. Motor nerve conduction (MNCV) was reduced to 21.6 m/s (±8.21, SD) in the median nerve, and sensory nerve action potentials were not detectable even in children.

Taken together, the clinical phenotype was homogeneous to previously reported series [2,3,22] and characterized by childhood-onset, severe and progressive sensory-motor neuropathy with a demyelinating pattern. In comparison with CMT4D (Lom type), the other recessive form of CMT exclusively found in the gypsy population, CMT4G patients were characterized by upper limb involvement at later ages, a less severe demyelinating pattern in MNCV, and no sign of deafness (Supplementary Table S1).

### 2.5. Mutated HK1 Is Unable to Interact with VDAC in PBMCs of CMT4G Patients

**Figure 5:**
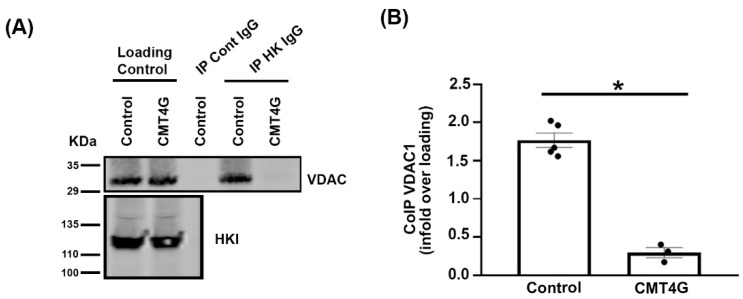
**HK-VDAC interaction is decreased in PBMCs of CMT4G patients.** (**A**) Representative Western blots showing HK1 and VDAC protein amount in PBMC extracts (loading control) from control patients (control) and CMT4G patients (CMT4G) and in immunoprecipitates with control rabbit immunoglobulins (IP Cont IgG) or with rabbit immunoglobulin directed against HK (IP HK IgG). VDAC Western blot shows that less VDAC coimmunoprecipitates with HK in PBMC extracts of CMT4G patient. (**B**) VDAC protein amount was quantified in immunoprecipitates of control patient or CMT4G patient using densitometry on Western blot. This amount was normalized to the amount of protein present in PBMC extracts. n = 2 independents experiments with 5 control patients and 3 CMT4G patients. Error bars show SEM and two-tailed Student *t*-test was performed. * *p* < 0.05; *p*-value = 3.6 × 10^–5^.

We collected PBMCs of three patients and control PBMCs were obtained from five individuals through the Etablissement Français du Sang of Montpellier. PBMC proteins were extracted, and HK was immunoprecipitated. Using Western blot, we found that VDAC co-precipitated with HK in PBMC extracts of control samples, while it was significantly less precipitated with HK in PBMC extracts of CMT4G patients (Figure 5), showing that CMT4G mutation in HK affects its interaction with VDAC.

**Figure 6:**
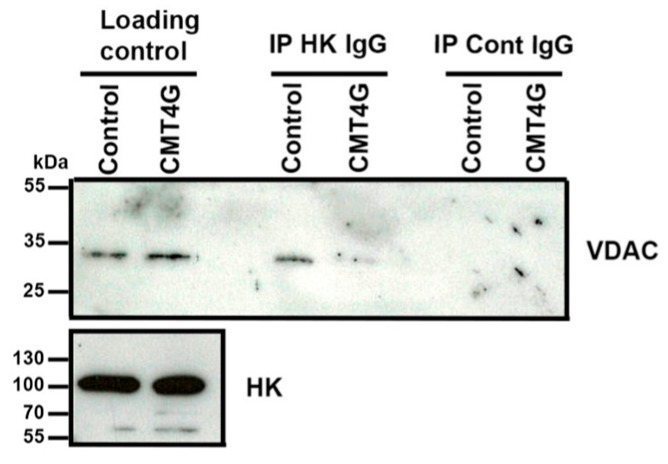
**HK-VDAC interaction is decreased in sural nerve of CMT4G patient.** Representative Western blots showing HK1 and VDAC protein amount in sural nerve extracts (loading control) from control patient (control) and CMT4G patient (CMT4G) and in immunoprecipitates with control rabbit immunoglobulins (IP Cont IgG) or with rabbit immunoglobulin directed against HK (IP HK IgG). VDAC Western blot shows that less VDAC coimmunoprecipitates with HK in sural nerve extracts of CMT4G patients. n = 1 patient and 1 control.

Further, we collected a sural nerve sample of one CMT4G patient and of a control patient. Proteins of these samples were extracted and immunoprecipitated with immunoglobulin directed against HK as described previously. Again, we found less VDAC coimmunoprecipitated with HK in the CMT4G patient nerve sample than in the control patient nerve sample (Figure 6), confirming data obtained in PBMCs.

Taken together, these data indicated that CMT4G patient nerve cells exhibit a defect in the interaction between HK1 and VDAC.

## 3. Discussion

CMT4G is a rare genetic peripheral neuropathy that heavily affects the quality of life of patients. Our new cohort of patients from the French gypsy community confirm that the disease presents with an early onset, a diffuse sensory and motor demyelinating neuropathy, and muscle wasting that leads them to become wheelchair-bound in their fifth decade. In 2009, a mutation in the 5′ non-coding sequence of the Hexokinase 1 gene was found to be the genetic cause of the disease [5]. However, the molecular grounds for this neuropathy remained unclear. From the data we present here, we propose that the CMT4G mutation leads to an alteration in the HK1 N-terminus. Unable to bind to VDAC, the mutant protein induces a sustained release of mitochondrial calcium in the cytoplasm, leaving mSC prone to demyelination. This mechanism may explain the demyelinating peripheral neuropathy that affects CMT4G patients.

However, some points remain to be clarified.

We propose that the alternative splicing of HK1 mRNA CMT4G mutation results in a modification of the 21 aa of the N-terminus of the protein sequence. This novel isoform is expected to have a similar molecular weight as the wt isoform. In addition, as the enzymatic activity requires a more distal region of the protein, no alteration of this activity is expected. This is consistent with the data presented in the literature. However, we were unable to purify the mutated protein in patient samples. Therefore, we did not confirm that the CMT4G mutant protein is indeed altered in its N-terminal sequence.

To understand if such a change modifies the ability of the protein to interact with VDAC, we expressed it in cells in culture. While the mutant protein expression and molecular size were similar to the wt protein, significantly less mutant protein interacted with VDAC. After 48 h of expression, the mutant protein decreased the mitochondrial calcium content versus the expression of wt protein. This is consistent with the role of HK as an inhibitor of the VDAC permeability to calcium: less interaction with VDAC leads to more release of calcium through this channel and a decrease in the mitochondrial calcium level. To confirm that the N-terminal of HK is responsible for this inhibitory effect on VDAC permeability to calcium, we used synthetic N-terminal peptides as inhibitors [16]. Methyl jasmonate was used to specifically remove endogenous HK from VDAC. We found that the N-terminal peptide of CMT4G mutant’s HK is unable to block mitochondrial calcium release through VDAC. Therefore, if this modification of the HK N-terminal is present in CMT4G patients' cells, then mitochondrial calcium homeostasis is going to be altered with less calcium in the mitochondria and more in the cytoplasm. A similar situation was observed in vivo in demyelinating Schwann cells following nerve injury [11] and in myelinating Schwann cells of diabetic mice [11] and resulted in myelin defects and peripheral neuropathy [11].

To check whether a similar situation is present in cells of CMT4G patients, we collected their PBMCs and investigated the interaction between HK and VDAC in these cells. Such as in cells expressing the mutant isoform of HK, while HK expression was similar to the expression in control PBMCs, a decreased interaction was found. In addition, we collected sural biopsies from a CMT4G patient and a control individual and coimmunoprecipitation showed that the HK protein of this patient’s nerve has a reduced ability to bind to VDAC. While this confirms data obtained from PBMCs, the number of samples is too low to draw a clear conclusion. Unfortunately, the collection of more biopsies was limited due to the invasive nature of the procedure. In addition, for technical reasons, we could not investigate the calcium homeostasis in cells of these biopsies.

Several other questions that we did not investigate remain open. Firstly, data on mRNA in CMT4G patient samples suggest that the alternative splicing resulting from the mutation is not complete and several copies of the wt mRNA are produced [5]. In what measure this leads to a co-expression of the mutant and wt protein in each cell remains undocumented. If both proteins are co-expressed in the same cell, then how the mutant protein affects the stability of the myelinated Schwann cell remains unclear. Does the mutant protein have a dominant negative effect on the wt protein function regarding its inhibition of VDAC permeability to calcium?

Secondly, we found that PBMCs of CMT4G patients show a significantly altered HK/VDAC interaction and that HEK293 cells with an altered HK/VDAC interaction leak mitochondrial calcium in the cytoplasm. Yet, no PBMC-related defects were ever reported in CMT4G patients. So, our present and previous data [11] suggest that the alteration of HK/VDAC interaction is only significantly relevant in mSC and not in other cells. Do these cells have a particular physiology that make them more sensitive to the weakness of HK/VDAC interaction and to the release of mitochondrial calcium?

Finally, we found that a peptide that mimics the HK1 N-terminal is able to prevent mitochondrial calcium release through VDAC. In a recent publication, we designed novel peptides bearing the same property with an improved efficacy and stability [16]. Some of these peptides prevent mouse nerve demyelination ex vivo. It is therefore possible that some of these peptides constitute a good basis for pharmacological treatment of CMT4G disease by preventing demyelination induced by the weakness of HK1 interaction with VDAC.

## 4. Materials and Methods

### 4.1. Study Design and Patient Series

Data from 25 patients affected by Russian-HMSN belonging to 9 unrelated gypsy families were collected by neurologists working at several hospitals in France. The genotype was obtained using Sanger analysis of the alternative AltT2 exon in the HK1 gene. A full neurological examination followed by blood sample collection was performed for all the patients. The genotype was obtained using Sanger analysis of the alternative AltT2 exon in the HK1 gene. Sural nerve biopsy was conducted in patient 10 while undergoing ankle arthrodesis. All patients were aware of the investigative nature of the studies and informed consent was obtained. The Montpellier University Hospital IRB reviewed and authorized this study (IRB-MTP_2021_04_202100783).

### 4.2. Plasmids and Reagents

HK peptides were dissolved in PBS (10 mM final concentration) and stored at −20 °C. Peptides were obtained from Proteomic Solutions (Saint Marcel, France). The sequences were as follows: HK1 peptide Ac-MIAAQLLAYYFTELKGRKKRRQRRRPPQ-NH2, HK1-mut peptide Ac-MGQICQRESATAAEKGRKKRRQRRRPPQ-NH2, and control peptide Ac-GRKKRRQRRRPPQ-NH2. Methyl jasmonate (final concentration 6 mM) was diluted in sterile PBS with 0.05% DMSO and 5% ethanol. Human wild-type (HK WT) and mutated (HK MUT) HKI cDNAs were cloned into a pCMV3 plasmid along with a Flag tag (Sino Biological, Beijing, China).

### 4.3. Cell Culture and Transfection

Human embryonic kidney (HEK)-293T cells were grown in Dulbecco’s modified Eagle’s medium (DMEM) supplemented with 2 mM L-glutamine, 100 U/mL penicillin/streptomycin, and 5% (*v*/*v*) heat-inactivated Fetal Bovine Serum (FBS) (all from Invitrogen, Waltham, USA). Cells were maintained at 37 °C in an atmosphere of 5% CO_2_, and passaged every 3 to 4 days.

HEK-293T cells in 6-well dishes or in 75 cm^2^ flasks were transfected using the jetPRIME in vitro DNA transfection kit (Polyplus Transfection, New York, NY, USA; Reference #114-15). For calcium imaging studies, cells were transfected with 0.35 µg of pAAV-mito-GCaMP2 or no plasmid. For coimmunoprecipitation studies, 1 µg of pCMV3-HKI WT or HKI MUT cells were transfected. A period of 48 h after transfection, mitochondrial calcium analysis was performed, or cells were lysed for the CoIP study.

### 4.4. PBMC Collection and Preparation

An amount of 100 mL of blood was obtained from the Etablissement Français du Sang of Montpellier (EFS). To isolate PBMCs, 20 mL of blood was gently layered over an equal volume of Ficoll in a Falcon tube and centrifuged for 30–40 min at 400–500 g. Four layers containing different cell types were formed. The first layer contained the plasma, which was discarded by pipetting. The second layer contained the PBMCs, which were sampled to perform the coimmunoprecipitation experiment. For the PBMC sampling, 1 mL of the layer was gently removed using a Pasteur pipette and added into 1 mL of fresh PBS. Then, the cell dilution was aliquoted in equal volumes and immediately frozen at −80 °C.

### 4.5. Immunoprecipitation

Transfected cells or PBMCs were lysed using 500 µL of lysis buffer (Tris HCl 0.5 M pH 8, NaCl 0.1 M, EDTA 2 mM, Triton 0.1%) containing a protease inhibitor mixture (1/1000, Sigma Aldrich, San Luis, MO, USA; Reference P8340). The cellular debris was removed by centrifugation at 13,000× *g* for 5 min at 4 °C and protein concentration was measured using the bicinchoninic acid method (Thermo Fisher, Waltham, MA, USA; Reference 23225) using bovine serum albumin dilutions as standard. The supernatant (10 mg/mL of protein) was processed for immunoprecipitation as described in the immunoprecipitation protocol for the Dynabeads Protein G kit (Invitrogen, Waltham, MA, USA) using 25 µg of mouse anti-flag antibody (1:100, Sigma Aldrich, Reference F9291-2MG) or 10 µg of mouse anti-HK antibody (1:100, Sigma Aldrich, Reference SAB1409191) for transfected cells and PBMCs, respectively. Immunoprecipitates were separated on denaturing 10% SDS-polyacrylamide gel and transferred onto nitrocellulose membranes. Membranes were blocked for 60 min with 5 mL of Licor Blocking Buffer TBS (Licor, Lincoln, NB, USA; Refence 927-60001). The following primary antibodies were incubated overnight at 4 °C in the same blocking buffer: rabbit anti-VDAC1 (1:100, Sigma Aldrich, Reference ZRB1315), mouse anti-flag (1:100, Sigma Aldrich, Reference F9291-2MG), or mouse anti-HK (1:100, Sigma Aldrich, Reference SAB1409191). Membranes were then washed 3 times for 10 min in TBS with Tween-20 (0.1% *v*/*v*) and then incubated for 1 h at room temperature with secondary antibodies: donkey anti-rabbit IRDye 800 (1:10.000, Licor, Reference 926-32213) and Goat anti-mouse IRDye 680 (1:10.000, Licor, Reference 926-68072). Membranes were then washed three times for 10 min each with TBS Tween-20. Visualization was performed using a Licor scanning device and quantification was performed using Image J software (version 4.0).

### 4.6. Immunocytochemistry

For immunocytochemistry studies, HEK-293T cells were seeded on polylysine-coated coverslips. Two days after transfection, cells were washed with PBS and fixed for 20 min in 2% paraformaldehyde (PFA, EuroMedex, Souffelweyersheim, France). Coverslips were then washed in PBS and incubated in the blocking solution (PBS, gelatin fish 5%, 0.1% Triton-20, 0.02% Azide) for 1 h at room temperature. This was followed by primary anti-flag antibody (1:100, Sigma-Aldrich) incubation in blocking solution for 90 min at 37 °C. After 4 washes with PBS, coverslips were incubated with mouse anti-mouse AlexaFluor 594 antibody (1:5000; Abcam, Cambridge, UK) for 1 h RT. Finally, DAPI staining was followed by mounting with DAKO fluorescence Mounting Medium (Agilent, Santa Clara, CA, USA). Images were acquired using a confocal LMS700 microscope (Zeiss, Rueil Malmaison, France) using a 63× lens (Zeiss, France) and analyzed using FIJI software (version 2.0.0-rc-68/1.52e). On a TIFF image, we quantified the integrated density of fluorescence after background subtraction. All experiments were performed in triplicates and repeated three times.

### 4.7. Videotracking Experiments

Mitochondrial calcium release was analyzed in HEK-293T cells transfected with pAAV-mitoGCaMP2 in a 35 mm FluoroDish (WPI-Europe, Friedberg, Germany; Reference FD35PDL). Two days after transfection, mitochondrial probe fluorescence was tracked for 46 min. Images were taken every 2 min using an Inversed Zeiss Axio-Observer microscope (Zeiss, France) with a live imaging setup to maintain temperature (37 °C) and hygrometry (CO_2_ 5.2%) (Incubation System S, Zeiss, France). An amount of 6 mM of methyl jasmonate diluted in PBS, DMSO 0.05%, and ethanol 5% was added with or without peptides (5 µM in PBS) at minute 12. Images were analyzed using Zen Blue 2.1 software (Zeiss, France). For each time point, we quantified the mean intensity value/area in 20 ROIs containing several cells by subtracting the background measured in empty areas of each ROI. These values were then normalized to the average basal staining before treating cells. The experiments were performed in duplicates and repeated three times.

### 4.8. Data and Statistical Analysis

Data are represented as mean ±SD according to the sample population size. Before statistical analysis, group normality was assessed using D’Agostino-Pearson. Statistical significances were determined using a two-tailed Student’s t test, one-way and two-way ANOVA, followed by a Bonferroni’s multiple comparison post hoc test. Significance was set at * *p* < 0.05, ** *p* < 0.01, or *** *p* < 0.001. ns indicates non-significant differences (*p* > 0.05). *n* indicates the number of independent experiments.

## Figures and Tables

**Table 1:** Clinical characterization of the French CMT4G cohort.

Id. # Patient	Age at Onset of LL Weakness (Years)	Age at Onset of UL Weakness (Years)	Foot Deformities	CMTNS	MNCV Median (m/s)	CMAP Median, (mV)	SNAP
**1**	6	16	+Arthrodesis	24	16.7 (38)	0.2	NR
**2**	6	15	+Arthrodesis	23	NR (29)	NR	NR
**3**	9	14	+	28	NR (65)	NR	NR
**4**	10	11	+ Arthrodesis	12	11.2 (10)	0.4	NR
**5**	7	ND	-	ND	ND	ND	ND
**6**	9	ND	+	ND	ND	ND	ND
**7**	7	16	+Arthrodesis	ND	ND	ND	ND
**8**	5	16	-	23	29 (9)	1.5	NR
**9**	9	16	-	19	16.2 (21)	1.4	NR
**10**	8	ND	+Arthrodesis	6	24.1 (10)	5.6	NR
**11**	3	ND	-	1	28.9 (3)	4.3	NR
**12**	4	ND	+	8	32 (9)	1.93	NR
**13**	12	35	-	25	ND	ND	ND
**14**	10	-	ND	-	-	-	-
**15**	3	-	ND	-	-	-	-
**16**	3	16	+Arthrodesis	16	21 (36)	0.59	NR
**17**	3	-	ND	-	-	-	-
**18**	10	10	+ Arthrodesis	17	23.8 (12)	-	NR
**19**	11	45	ND	ND	ND	ND	ND
**20**	13	20	+Arthrodesis	-	-	-	-
**21**	6	ND	-	6	30.6 (7)	5.3	NR
**22**	10	14	+Arthrodesis	ND	22.2 (8)	1.19	NR
**23**	5	ND	-	ND	24 (6)	6.4	NR
**24**	4	7	-	20	16 (27)	0.45	NR
**25**	12	8	-	ND	ND	ND	ND

CMAP: compound motor action potential; CMTNS: Charcot–Marie–Tooth Neuropathy Score; MNCV: motor nerve conduction velocity; NR: no response; ND: not done; SNAP: sensory nerve action potential.

## Data Availability

All non-patient-related data generated or analyzed during this study are included in this published article and its Supplementary Information Files.

## Supplementary Materials

The following supporting information can be downloaded at https://www.mdpi.com/article/10.3390/ijms25084364/s1.

## References

[1] Gabrikova D., Mistrik M., Bernasovska J., Bozikova A., Behulova R., Tothova I., Macekova S. (2013). Founder mutations in NDRG1 and HK1 genes are common causes of inherited neuropathies among Roma/Gypsies in Slovakia. J. Appl. Genet..

[2] Sevilla T., Martínez-Rubio D., Márquez C., Paradas C., Colomer J., Jaijo T., Millán J., Palau F., Espinós C. (2013). Genetics of the Charcot-Marie-Tooth disease in the Spanish Gypsy population: The hereditary motor and sensory neuropathy-Russe in depth. Clin. Genet..

[3] Brožková D., Haberlová J., Mazanec R., Laštůvková J., Seeman P. (2016). HSMNR belongs to the most frequent types of hereditary neuropathy in the Czech Republic and is twice more frequent than HMSNL. Clin. Genet..

[4] Thomas P.K., Kalaydjieva L., Youl B., Rogers T., Angelicheva D., King R.H.M., Guergueltcheva V., Colomer J., Lupu C., Corches A. (2001). Hereditary motor and sensory neuropathy-russe: New autosomal recessive neuropathy in balkan gypsies. Ann. Neurol..

[5] Hantke J., Chandler D., King R., Wanders R.J., Angelicheva D., Tournev I., McNamara E., Kwa M., Guergueltcheva V., Kaneva R. (2009). A mutation in an alternative untranslated exon of hexokinase 1 associated with hereditary motor and sensory neuropathy—Russe (HMSNR). Eur. J. Hum. Genet..

[6] Rosa J.C., César M.d.C. (2016). Role of Hexokinase and VDAC in Neurological Disorders. Curr. Mol. Pharmacol..

[7] Bianchi M., Crinelli R., Serafini G., Giammarini C., Magnani M. (1997). Molecular bases of hexokinase deficiency. Biochim. Biophys. Acta (BBA)-Mol. Basis Dis..

[8] Peters L.L., Lane P.W., Andersen S.G., Gwynn B., Barker J.E., Beutler E. (2001). Downeast Anemia (dea), a New Mouse Model of Severe Nonspherocytic Hemolytic Anemia Caused by Hexokinase (HKI) Deficiency. Blood Cells Mol. Dis..

[9] Galluzzi L., Kepp O., Tajeddine N., Kroemer G. (2008). Disruption of the hexokinase–VDAC complex for tumor therapy. Oncogene.

[10] Camara A.K.S., Zhou Y., Wen P.-C., Tajkhorshid E., Kwok W.M. (2017). Mitochondrial VDAC1: A Key Gatekeeper as Potential Therapeutic Target. Front. Physiol..

[11] Tricaud N., Gautier B., Berthelot J., Gonzalez S., Van Hameren G. (2022). Traumatic and Diabetic Schwann Cell Demyelination Is Triggered by a Transient Mitochondrial Calcium Release through Voltage Dependent Anion Channel 1. Biomedicines.

[12] Shoshan-Barmatz V., Maldonado E.N., Krelin Y. (2017). VDAC1 at the crossroads of cell metabolism, apoptosis and cell stress. Cell Stress.

[13] Clapham D.E. (2007). Calcium Signaling. Cell.

[14] Bryan N., Raisch K.P. (2015). Identification of a mitochondrial-binding site on the N-terminal end of hexokinase II. Biosci. Rep..

[15] Magrì A., Belfiore R., Reina S., Tomasello M.F., Di Rosa M.C., Guarino F., Leggio L., De Pinto V., Messina A. (2016). Hexokinase I N-terminal based peptide prevents the VDAC1-SOD1 G93A interaction and re-establishes ALS cell viability. Sci. Rep..

[16] Gautier B., Jacquard M.F., Guelfi S., Abbou S., Gonzalez E., Berthelot J., Boukhaddaoui H., Lebrun A., Legrand B., Tricaud N. (2022). Mapping the N-Terminal Hexokinase-I Binding Site onto Voltage-Dependent Anion Channel-1 to Block Peripheral Nerve Demyelination. J. Med. Chem..

[17] Cohen S., Flescher E. (2009). Methyl jasmonate: A plant stress hormone as an anti-cancer drug. Phytochemistry.

[18] Goldin N., Arzoine L., Heyfets A., Israelson A., Zaslavsky Z., Bravman T., Bronner V., Notcovich A., Shoshan-Barmatz V., Flescher E. (2008). Methyl jasmonate binds to and detaches mitochondria-bound hexokinase. Oncogene.

[19] Cesari I.M., Carvalho E., Rodrigues M.F., Mendonça B.d.S., Amôedo N.D., Rumjanek F.D. (2014). Methyl Jasmonate: Putative Mechanisms of Action on Cancer Cells Cycle, Metabolism, and Apoptosis. Int. J. Cell Biol..

[20] Chen M., Wang Y., Hou T., Zhang H., Qu A., Wang X. (2011). Differential mitochondrial calcium responses in different cell types detected with a mitochondrial calcium fluorescent indicator, mito-GCaMP2. Acta Biochim. Biophys. Sin..

[21] Tallini Y.N., Ohkura M., Choi B.R., Ji G., Imoto K., Doran R., Lee J., Plan P., Wilson J., Xin H.-B. (2006). Imaging cellular signals in the heart in vivo: Cardiac expression of the high-signal Ca^2+^ indicator GCaMP2. Proc. Natl. Acad. Sci. USA.

[22] Guergueltcheva V., Tournev I., Bojinova V., Hantke J., Litvinenko I., Ishpekova B., Shmarov A., Petrova J., Jordanova A., Kalaydjieva L. (2006). Early Clinical and Electrophysiologic Features of the Two Most Common Autosomal Recessive Forms of Charcot-Marie-Tooth Disease in the Roma (Gypsies). J. Child Neurol..

