# Supplementary material for: The Hexokinase 1 5′-UTR Mutation in Charcot–Marie–Tooth 4G Disease Alters Hexokinase 1 Binding to Voltage-Dependent Anion Channel-1 and Leads to Dysfunctional Mitochondrial Calcium Buffering
Source: Int J Mol Sci. 2024 Apr 15;25(8):4364. doi: 10.3390/ijms25084364 (PMC11050395; doi:10.3390/ijms25084364)
Supplement: Supplementary file 1 [file ijms-25-04364-s001.zip › ijms-2890356-supplementary.pdf]

Table S1

	CMT (Lom)	CMT4G	CMT4C
**Mutated gene**	**NDRG1**<br>- p.R148X in gipsy population<br>- Duplication of exons 6-8 in Turkey	**HK1**<br>g.9712C	**SH3TC2**<br>- Many variants reported<br>- p.Arg1109X and p.C737_P738delinsX are exclusive of gypsy population
**Epidemiology**	- Gypsy population from Spain, Slovenia, Bulgaria and Italy<br>-Turkey	- Gypsy population from Spain<br>- Slovakia and Bulgaria	Mediterranean countries
**Mean age of onset (yr)**	6.7	11	Variable
**Age of upper limb involvement (yr)**	12.7	22	Variable
**Evolution**	Wheelchair bound by the age >25-40 yr	Wheelchair bound by the age of >50 yr	Variable
**Other clinical signs**	Deafness, lingual atrophy	-	Deafness, lingual atrophy
**Median nerve NCV (m/s)**	9.6	20-32	13-30 with C. block
**SNAP**	Absent	Absent	Absent
**Biopsy**	Onion bulbs	Clusters of regeneratives fibers	Onion bulbs

yr: years; CMAP: compound motor action potential; CMTNS: Charcot-Marie-Tooth Neuropathy Score; MNCV: motor nerve conduction velocity; NR: not response; ND: Not done; SNAP: sensory nerve action potential
